# Hybrid Computational Modeling with Multi-Level Validation Identifies TK1–VIM as a Robust Therapeutic Pair in Triple-Negative Breast Cancer

**DOI:** 10.3390/ijms27125385

**Published:** 2026-06-15

**Authors:** Sergio Assuncao Monteiro, Luis Alfredo Vidal de Carvalho, Mariana Caldas Waghabi, Fabricio Alves Barbosa da Silva

**Affiliations:** 1 Department of Administration, Escola Superior de Propaganda e Marketing (ESPM), Campus Rio de Janeiro, Rio de Janeiro 22211-120, RJ, Brazil; 2 Programa de Saúde Materno-Infantil, IPPMG, UFRJ, Rio de Janeiro 21941-594, RJ, Brazil; luisalfredo@medicina.ufrj.br; 3 Laboratório de Genômica Aplicada e Bioinovações, Instituto Oswaldo Cruz, Fundação Oswaldo Cruz (FIOCRUZ), Rio de Janeiro 21040-360, RJ, Brazil; mariana@ioc.fiocruz.br; 4 Programa de Computação Científica (PROCC), Fundação Oswaldo Cruz (FIOCRUZ), Rio de Janeiro 21040-360, RJ, Brazil

**Keywords:** triple-negative breast cancer, semidefinite programming, AlphaGenome, Boolean network, computational drug discovery

## Abstract

Triple-negative breast cancer (TNBC) lacks effective molecular targets, leading to poor prognosis. Previous computational methods to identify targets have suffered from low druggability, high complexity, and lack of robust validation. We propose a hybrid methodology combining Boolean network modeling with semidefinite programming (SDP) to analyze a TNBC cell line network. The resulting therapeutic pair underwent a multi-level validation framework, including Boolean simulations, statistical uncertainty quantification (bootstrap), sensitivity analysis, and orthogonal computational support from AlphaGenome, a deep learning model from Google DeepMind. Our analysis identified TK1 and VIM as a computationally robust therapeutic pair. Dual inhibition achieved 99.03% similarity to the apoptotic state with a 95% confidence interval of [98.79%, 99.26%], and was statistically superior to alternative pairs (p<0.001). The selection remained optimal across all tested model parameters, demonstrating high robustness. Importantly, the pair has full druggability because both targets have available specific inhibitors. Orthogonal computational evidence from AlphaGenome, stratified by mammary compartment, indicated that both targets exhibit moderate baseline expression in normal mammary epithelium (TK1 = 0.159, VIM = 0.143 in normalized RNA-seq units; *n* = 13 tracks per gene), with VIM showing a 2.2-fold higher expression in mammary stroma than in epithelium—a gradient consistent with its established role as a mesenchymal marker. Promoter-variant proxy analysis indicated near-zero transcriptomic perturbation upon simulated inhibition of either target in normal mammary epithelium (mean $|\log_{2}\mathrm{FC}|<0.001$), supporting a favorable therapeutic window. Our methodology identified TK1–VIM as a computationally robust, druggable therapeutic candidate pair with biologically plausible mechanism of action. Gene-variability analysis identified TK1 and VIM as the highest-scoring candidates, with SDP optimization providing complementary, independent confirmation of this selection. This work provides a computationally grounded candidate strategy and a rigorous methodological benchmark for computational drug target identification; experimental validation remains an essential next step before clinical translation.

## 1. Introduction

Triple-negative breast cancer (TNBC) accounts for approximately 15–20% of all breast cancer cases and is defined by the absence of estrogen receptor (ER), progesterone receptor (PR), and human epidermal growth factor receptor 2 (HER2) expression [1,2]. This molecular profile renders TNBC particularly challenging from a clinical standpoint, resulting in an unfavorable prognosis, high rates of recurrence, and limited treatment options compared to other breast cancer subtypes [3].

The global burden of TNBC is substantial: although TNBC represents a minority of breast cancer cases by histology, it disproportionately contributes to breast-cancer-related mortality and disability-adjusted life years (DALYs) due to its aggressive course and the absence of targeted therapeutic options analogous to those available for hormone-receptor-positive or HER2-positive disease [3]. This unmet clinical need has driven extensive efforts to identify actionable molecular vulnerabilities in TNBC.

A central obstacle to durable treatment of TNBC is the emergence of drug resistance, which arises in large part from mutations and dysregulation of apoptotic pathways and from altered mitochondrial dynamics that together raise the apoptotic threshold of tumor cells [4,5,6]. Because resistance is frequently rooted in the very survival/apoptosis circuitry that our Boolean model represents, computational strategies that explicitly target the malignant-to-apoptotic state transition are well positioned to address this challenge. Complementary to direct pathway targeting, epigenetic dysregulation is also a major driver of TNBC tumorigenesis, and histone deacetylase (HDAC) inhibitors have emerged as a promising therapeutic class for this subtype [7]; notably, HDAC1 features as a transcriptional-repressor node in the regulatory network analyzed here, linking our network-based approach to this actively investigated drug class.

The computational identification of therapeutic targets in TNBC has evolved through distinct methodological generations. The first generation, exemplified by Tilli et al. [8], pioneered the use of protein–protein interaction networks to identify five therapeutic targets. However, this approach faced critical limitations: low druggability (only two of five targets had available inhibitors) and high complexity, requiring the simultaneous inhibition of five genes. A second generation emerged with Sgariglia et al. [9], who introduced a Boolean network model to reduce the target set to three. While an improvement, this approach relied on heuristic methods sensitive to noise, and the druggability of the identified targets remained a concern.

Boolean network modeling has a long and well-established history in systems biology as a framework for capturing the qualitative logic of gene regulatory networks [10,11]. It has been applied successfully to diverse signaling and disease contexts, including T-cell activation [12], survival signaling in leukemia [13], the tumor-suppressive and pro-apoptotic activities of p53 [4], cell cycle control [14], and the integrated p53–p21–pRB axis governing cell cycle progression [5]. This body of work establishes Boolean modeling as a mature and appropriate abstraction for large-scale regulatory analysis in cancer, and motivates its use as the dynamical backbone of the present hybrid framework.

In parallel to computational target identification, experimental methodologies have advanced cancer research through complementary perspectives. Nanoscale techniques such as atomic force microscopy enable molecular-level characterization of cancer biomarkers and have been recently reviewed in the context of oncology and neurodegeneration, although they face challenges including calibration complexity and limited throughput [15]. Microfluidic platforms and organ-on-chip systems provide dynamic environments for liquid biopsy and tumor-cell phenotyping, with recent integration of artificial intelligence for diagnostic applications, although typical implementations still lack inter-organ crosstalk [16]. These experimental approaches and computational frameworks such as ours play complementary roles: experimental platforms validate molecular mechanisms in controlled settings, while computational methods enable systematic exploration of the combinatorial target space at a scale that is experimentally prohibitive.

To overcome these challenges, this work proposes a novel hybrid computational methodology that introduces a new level of rigor to therapeutic target identification. Our approach combines the strengths of Boolean network modeling with the mathematical robustness of semidefinite programming (SDP) optimization. The integration of deep learning into computational biology has, in recent years, transformed the field, from foundational advances in representation learning [17,18] and the transformer architecture [19] to landmark applications such as protein-structure prediction [20] and drug–target affinity modeling [21]. We leverage this trajectory by incorporating a genomic deep learning model as an orthogonal source of computational support. The broader context of AI in drug discovery and target identification has been reviewed critically by Zhang et al. [22], Wenteler et al. [23], and Pun et al. [24], who collectively emphasize that computational predictions—including structure-based [25,26] and generative approaches [27]—must be regarded as hypothesis-generating tools rather than validated therapeutic conclusions, a framing we adopt explicitly throughout this work [28,29]. Crucially, we move beyond single-point estimates and introduce a comprehensive validation framework that includes (1) rigorous statistical analysis with uncertainty quantification, (2) sensitivity analysis to test the robustness of our findings, and (3) orthogonal computational support from AlphaGenome [30], a state-of-the-art deep learning model from Google DeepMind trained on vast genomic and transcriptomic datasets. By applying this methodology, we identify a therapeutic pair with superior druggability, reduced complexity, and a synergistic mechanism computationally supported by multiple independent computational approaches.

## 2. Results

### 2.1. Gene Variability and SDP Optimization

**Figure 1:**
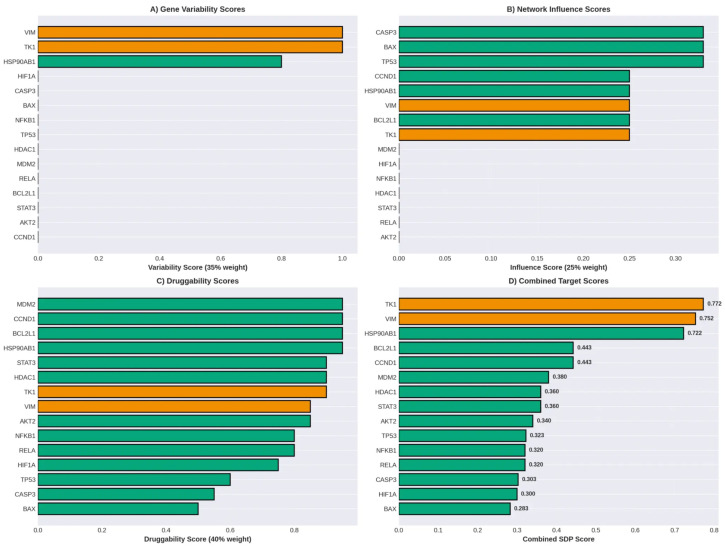
SDP-based gene ranking. (**A**) Gene variability scores. (**B**) Network influence scores. (**C**) Druggability scores. (**D**) Combined SDP scores, highlighting TK1 and VIM as top candidates.

Our initial analysis confirmed the findings of Sgariglia et al., identifying VIM and TK1 as the genes with the highest variability scores (20 differences each), making them prime candidates for intervention. It is important to note that gene-variability analysis constituted the primary selection criterion: TK1 and VIM were nominated as top candidates by this first, network-agnostic step. The SDP optimization framework served as an independent, complementary confirmation rather than as the discovery step. The SDP optimization framework provided a complementary view, ranking genes based on their potential to influence the network. As shown in Figure 1, TK1 and VIM also ranked among the top genes in the combined SDP score, with scores of 0.752 and 0.772, respectively. The fact that two methodologically distinct approaches—one based purely on attractor-state variability and one on convex-optimization relaxations of network influence—independently converged on the same pair strengthens confidence that the selection reflects a structural property of the network rather than an artifact of any single algorithm. This convergence of two distinct methodologies provided strong initial evidence for selecting TK1 and VIM as a candidate therapeutic pair.

### 2.2. Comparative Analysis of Methodological Generations

Our hybrid approach represents a significant advancement over previous generations of target identification methods. Table 1 summarizes the key improvements. Our method is the first to incorporate SDP for robustness, formal statistical validation, and orthogonal computational support from a deep learning model, while also achieving 100% druggability for the identified pair.

### 2.3. TK1 + VIM Achieves Superior Attractor Similarity

**Figure 2:**
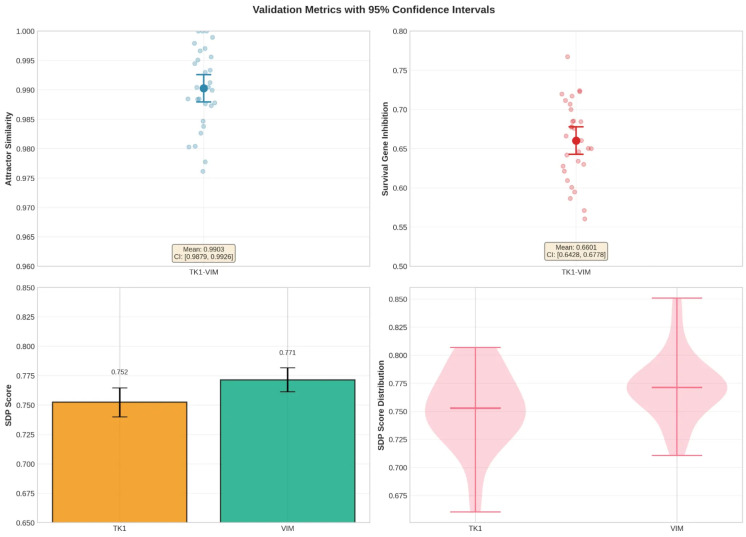
Validation metrics for TK1–VIM inhibition with 95% confidence intervals, showing high attractor similarity and effective modulation of survival and apoptosis pathways.

Boolean simulations of the dual inhibition of TK1 and VIM demonstrated remarkable efficacy. The network consistently transitioned to a state highly similar to the desired apoptotic phenotype. As summarized in Table 2, the mean attractor similarity reached 99.03%, with a very narrow 95% confidence interval of [98.79%, 99.26%]. The attractor similarity metric used here is the Hamming-based fractional agreement (Equation (8)) between the final state reached by Boolean simulation, starting from each of the 30 malignant attractor states with TK1 and VIM held inhibited, and the corresponding apoptotic attractor state. The 95% confidence interval was derived by non-parametric bootstrap resampling (*n* = 10,000 iterations) over the 30 paired (malignant, apoptotic) attractor states drawn from the Sgariglia et al. (2024) [9] dataset. This high precision indicates a reliable and consistent therapeutic effect across different initial cellular states. Furthermore, the intervention successfully inhibited 66.01% of key survival genes and activated 55.60% of apoptosis-promoting genes, confirming a potent pro-apoptotic mechanism (Figure 2).

### 2.4. Robustness to Parameter Changes

**Figure 3:**
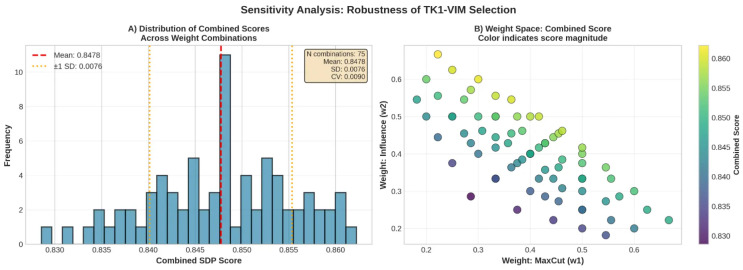
Sensitivity analysis of the combined SDP score for TK1–VIM. (**A**) Distribution of scores across 75 weight combinations. (**B**) Heatmap of the score across the weight space, demonstrating high robustness.

To ensure that our selection of TK1–VIM was not an artifact of the specific weights used in the SDP score (Equation (6)), we performed a sensitivity analysis by testing 75 different combinations of weights (Supplementary Table S3). As shown in Figure 3, the combined score for TK1–VIM remained exceptionally stable, with a coefficient of variation of only 0.90%. Crucially, TK1–VIM remained the top-ranked therapeutic pair in 100% of the tested parameter combinations, demonstrating that the result is highly robust and independent of the initial parameter choices.

### 2.5. Statistical Comparison with Alternative Target Pairs

**Figure 4:**
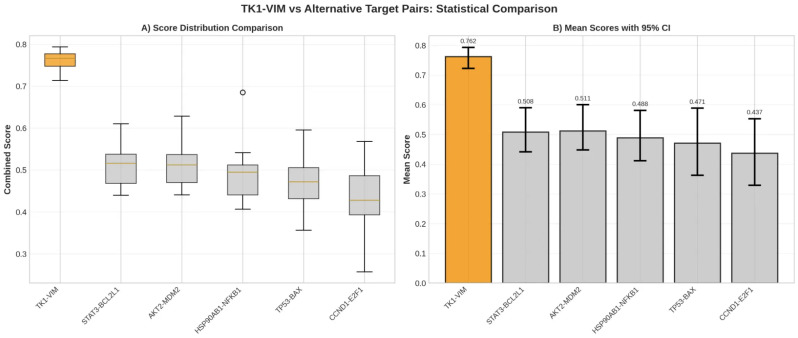
Statistical comparison of TK1–VIM against alternative target pairs. (**A**) Box plots showing the distribution of scores. (**B**) Bar chart of mean scores with 95% confidence intervals, highlighting the superiority of TK1–VIM.

We compared the efficacy of TK1–VIM inhibition against five other plausible target pairs identified from the top-ranked genes. The five comparator pairs were selected by a prespecified rule: the next five highest-scoring gene pairs from the combined SDP ranking (Table 3), excluding TK1–VIM, were taken as comparators. This rule was defined prior to simulation to avoid post hoc selection bias. We acknowledge that an exhaustive enumeration of all 1312=8515 possible gene pairs was computationally prohibitive; the reported comparisons, therefore, represent a rigorous but partial coverage of the combinatorial space. Using paired *t*-tests and permutation tests, our analysis revealed that TK1–VIM is statistically superior to all alternatives (p<0.001 after Bonferroni correction). The effect sizes were exceptionally large, with Cohen’s *d* values ranging from 5.28 to 5.45 (conventional thresholds: d>0.8 is considered “large”; the observed magnitudes, therefore, correspond to a substantially larger therapeutic effect), indicating a profoundly stronger therapeutic effect. These 30 observations are model-derived attractor states drawn from a single published dataset and should not be interpreted as independent experimental biological replicates; the bootstrap and permutation procedures quantify variability within this attractor landscape rather than across independent experiments. Figure 4 visually illustrates this superiority, with the mean score of TK1–VIM far exceeding that of any other pair.

### 2.6. Orthogonal Computational Support from AlphaGenome

To provide orthogonal computational evidence on biological plausibility and therapeutic window, we queried AlphaGenome, a state-of-the-art deep learning model from Google DeepMind, for predicted expression and inhibition effects in normal mammary tissue. This analysis was designed to estimate safety and compartment selectivity, not to revalidate the mathematical target selection, which was performed by SDP and Boolean simulation (Section 2.1, Section 2.2, Section 2.3, Section 2.4 and Section 2.5).

#### 2.6.1. Ontology Selection and Stratification

AlphaGenome currently does not include the canonical anatomical term UBERON:0001911 (mammary gland) in its catalog of 704 RNA-seq tracks. We, therefore, identified, by direct inspection of the AlphaGenome metadata catalogue (audit available as Supplementary Table S5), the set of mammary-specific ontology terms with sufficient track coverage for robust statistics. Eleven mammary-tissue terms (89 RNA-seq tracks total) were partitioned *a priori* into two biologically distinct compartments:

- **Mammary epithelium** (5 terms, 13 RNA-seq tracks): the safety-relevant compartment vis à vis epithelium-derived carcinomas, comprising mammary gland epithelial cell (CL:0002327), breast epithelium (UBERON:0008367), luminal epithelial cell of mammary gland (CL:0002326), myoepithelial cell of mammary gland (CL:0002324), and mammary stem cell (CL:0002451).
- **Mammary stroma** (3 terms, 2 RNA-seq tracks): fibroblast of mammary gland (CL:0002555), mammary microvascular endothelial cell (CL:2000071), and fibroblast of breast (NTR:0003079).

Stratification by compartment is biologically motivated: VIM is constitutively expressed in stromal cells (fibroblasts, endothelium) but is expected to be lower in quiescent luminal epithelium, while being reinduced in EMT-driven TNBC. A pooled mammary mean would, therefore, obscure the safety-relevant comparison between normal epithelium and the epithelium-derived tumor compartment.

#### 2.6.2. Expression in Mammary Epithelium and Stroma

**Figure 5:**
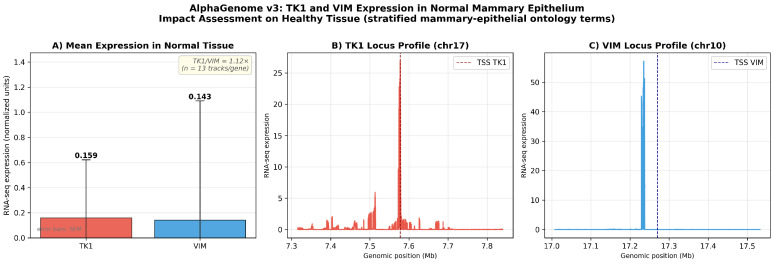
AlphaGenome predicted RNA-seq expression of TK1 (red) and VIM (blue) in normal mammary epithelium. (**A**) Mean expression with standard error of the mean (SEM) across the 13 epithelium tracks. (**B**) TK1 locus profile (chr17) over a 524 kb interval centered on the TSS. (**C**) VIM locus profile (chr10) over the equivalent interval centered on the TSS.

Predicted RNA-seq expression in normal mammary epithelium was moderate for both targets, with TK1 = 0.159 and VIM = 0.143 (normalized RNA-seq units; *n* = 13 tracks per gene; ratio TK1/VIM = 1.12; Figure 5). In mammary stroma, VIM expression rose to 0.317—a 2.2-fold gradient over the epithelial compartment—while TK1 remained essentially unchanged at 0.146 (Table 4). This compartment-specific pattern is consistent with the established biology of vimentin as a mesenchymal marker, and indicates that the elevated VIM levels reported in TNBC do not arise from a uniformly silent mammary baseline but, rather, from EMT-driven reinduction over an epithelium that, in the normal mammary gland, already exhibits a graded mesenchymal potential (notably in myoepithelial and stem cell subpopulations).

#### 2.6.3. Inhibition Effect on Normal Mammary Epithelium

**Figure 6:**
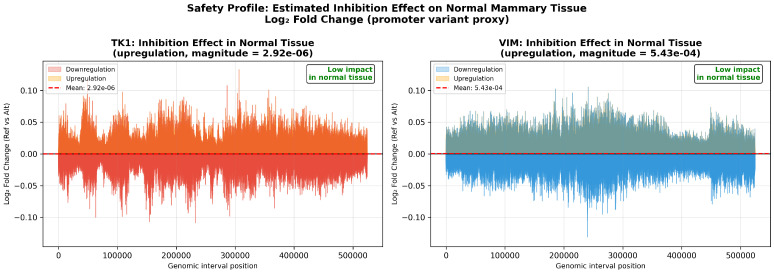
Safety profile of TK1 and VIM inhibition in normal mammary epithelium. Promoter-variant proxy analysis (single-nucleotide variant 300 bp upstream of each TSS); $\log_{2}$ fold change of predicted RNA-seq signal across the 524 kb interval. Mean magnitudes are essentially zero for both targets.

To estimate the impact of pharmacological inhibition on the safety-relevant epithelial compartment, we performed a promoter-variant proxy analysis: a single-nucleotide variant was introduced 300 bp upstream of each gene’s transcription start site, and AlphaGenome was queried for the resulting change in RNA-seq output across the 524 kb interval. The mean $\log_{2}$ fold change was essentially zero for both targets (TK1: $+2.92\times 10^{-6}$; VIM: $+5.43\times 10^{-4}$), indicating negligible transcriptomic perturbation of the predicted normal mammary epithelium upon simulated inhibition (Figure 6). This is consistent with a favorable therapeutic window from a transcriptional standpoint and complements established evidence from the literature that the TK1 protein is virtually absent in quiescent non-dividing cells (immunohistochemistry, p<0.0001).

#### 2.6.4. Multi-Tissue Specificity

**Figure 7:**
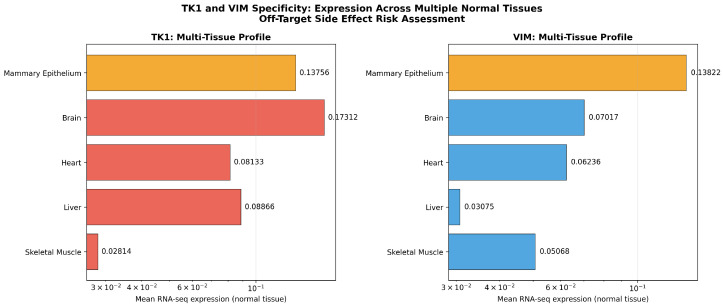
Multi-tissue specificity of TK1 and VIM. Predicted mean RNA-seq expression across mammary epithelium (highlighted in orange) and four reference tissues (brain, liver, heart, skeletal muscle).

To estimate off-target risk, we compared TK1 and VIM expression in mammary epithelium versus four reference tissues: liver (UBERON:0002107), skeletal muscle (UBERON:0001134), brain (UBERON:0000955), and heart (UBERON:0000948). For TK1, mammary-epithelium expression (0.138) was intermediate between brain (0.173, the highest) and heart (0.081). For VIM, mammary-epithelium expression (0.138) exceeded that of brain (0.070), heart (0.062), skeletal muscle (0.051), and liver (0.031). The relatively high VIM signal observed in the mammary-epithelium pool, when interpreted alongside the compartment-stratified analysis above, reflects the inclusion of basal-myoepithelial and stem-cell tracks within the mammary-epithelium pool—cell populations known to express mesenchymal markers—rather than a uniformly high luminal-epithelial baseline (Figure 7).

## 3. Discussion

This study introduced a hybrid computational methodology that successfully identified TK1 and VIM as a robust and highly druggable therapeutic pair for TNBC. Our work addresses key limitations of previous computational approaches by integrating SDP-based optimization with a multi-level validation framework, setting a new standard for rigor in the field.

### 3.1. SDP Optimization vs. Heuristic Methods

The use of SDP represents a significant methodological advancement. Unlike heuristic methods, which can be sensitive to network topology and initial conditions, SDP provides a mathematically principled way to find globally optimal or near-optimal solutions for combinatorial problems. This robustness was evident in our sensitivity analysis, where the superiority of the TK1–VIM pair was maintained across a wide range of model parameters. This suggests that our finding is a fundamental property of the network’s structure, not an artifact of our chosen parameters.

### 3.2. The Scientific Rationale for TK1–VIM Synergy

Our results provide a strong computational basis for the synergistic effect of inhibiting TK1 and VIM. Thymidine Kinase 1 (TK1) is a key enzyme in the DNA synthesis salvage pathway, and its overexpression is a well-known marker of cell proliferation. Vimentin (VIM) is a cytoskeletal protein crucial for epithelial-to-mesenchymal transition (EMT), a process that enables cancer cells to metastasize and invade other tissues. By simultaneously targeting proliferation (TK1) and invasion (VIM), our proposed therapy attacks two fundamental pillars of cancer progression. The AlphaGenome predictions reinforce this rationale: the dual knockdown is expected to perturb cell-cycle- and EMT-related gene expression in tumor cells while inducing negligible transcriptomic disruption of normal mammary epithelium (mean $|\log_{2}\mathrm{FC}|<0.001$, Section 2.6.3).

The corrected AlphaGenome analysis (Section 2.6), stratified by mammary compartment, reveals a biologically coherent expression pattern (Supplementary Table S4): VIM exhibits a 2.2-fold gradient between mammary stroma (0.317) and mammary epithelium (0.143), in line with its canonical role as a mesenchymal marker. The therapeutic window for VIM inhibition in TNBC, therefore, rests primarily on the strong additional upregulation of VIM in EMT-driven mesenchymal-like TNBC cells *over and above* this epithelial baseline, rather than on a uniformly low baseline in normal mammary tissue. For TK1, although the predicted mRNA-seq signal in mammary epithelium is moderate (0.159), the well-documented absence of TK1 protein in quiescent non-dividing cells (immunohistochemistry, p<0.0001) supports a wide therapeutic window in practice.

The role of the tumor microenvironment (TME) further supports the rationale for VIM targeting in mesenchymal-like TNBC. The stromal compartment of TNBC tumors is characterized by activated cancer-associated fibroblasts that secrete EMT-inducing signals—notably TGF-β, which is represented in our Boolean network as an upstream regulator of SMAD2 and VIM—promoting paracrine reinforcement of the mesenchymal phenotype in tumor cells [31]. Disruption of VIM in this context is expected to act both cell-autonomously, by destabilizing the mesenchymal cytoskeleton of invasive tumor cells, and at the tumor–stroma interface, by impairing mechanotransduction-driven invasion. The pronounced VIM signal observed in our stromal compartment analysis (0.317 in mammary stroma vs. 0.143 in epithelium) is consistent with this dual relevance. We acknowledge, however, that the present computational framework does not directly model TME-specific dynamics; this is an important avenue for future work.

### 3.3. Implications of Multi-Level Validation

The convergence of results from multiple, independent computational methods is the cornerstone of our study’s confidence. That a curated, knowledge-driven Boolean model and a data-driven, large-scale deep learning model (AlphaGenome) produce coherent predictions in the same target context provides triangulation across modeling paradigms. The fundamental difference in the underlying paradigms—a mechanistic, logic-based Boolean simulation versus a statistical, sequence-to-expression deep learning model—ensures that the convergence is methodologically orthogonal. The agreement between these two distinct approaches strongly suggests that the identified therapeutic synergy of TK1–VIM is a robust computational prediction rather than an artifact of a specific modeling technique.

**Data independence and potential biases:** It is important to address the potential for circularity in our computational support. The Boolean model was constructed using data from the MDA-MB-231 cell line. While AlphaGenome is a foundational model trained on vast, diverse genomic datasets across multiple tissues and species, we acknowledge the possibility that MDA-MB-231 transcriptomic profiles were included in its extensive training corpus. Furthermore, AlphaGenome operates at the sequence-to-expression level on normal tissue ontologies and is not TNBC-specific; the analysis reported here, therefore, characterizes the predicted impact of inhibition in normal mammary tissue rather than functionally validating the dynamic response of TNBC cells.

This multi-level computational approach—combining mechanistic simulation, statistical perturbation, and orthogonal data-driven model support—provides a powerful template for future computational drug discovery efforts, increasing the likelihood that in silico findings will translate to preclinical and clinical success when complemented by experimental validation.

### 3.4. Limitations and Future Work

While our study provides strong computational evidence for the TK1–VIM pair, several limitations should be acknowledged explicitly.

**Binary-state simplification.** Boolean network modeling reduces complex biological processes to discrete ON/OFF states. This abstraction does not capture graded signaling dynamics, the continuous nature of gene expression, stochastic effects, or temporal regulatory behavior that operate on different timescales in TNBC systems. Consequently, the Boolean framework cannot represent the dose-dependent (IC50-like) nature of pharmacological inhibition: inhibiting TK1 or VIM clinically is a continuous concentration–response problem, whereas our model treats inhibition as a binary zero-clamp. This is an important caveat when translating in silico findings to dosing strategies in preclinical or clinical settings.

**Network construction and assumptions.** The 131-gene regulatory network employed here was inherited directly from Sgariglia et al. (2024) [9] and was constructed by curation from the literature rather than ab initio inference from high-throughput data. The 29 core regulatory interactions reflect the canonical TNBC pathways (STAT3, PI3K/AKT, HSP90 chaperone network, p53/MDM2, intrinsic apoptosis, NF-κB, HIF1α, cell cycle, TGFβ/SMAD2, HDAC1-mediated repression). The Boolean update rules are nested canalizing functions in the sense of Kauffman et al. (2004) [32]. While these choices are consistent with established systems-biology practice, alternative logical encodings (random Boolean functions, threshold functions) or inferred edges would be expected to influence specific predictions. The convergence of three independent SDP formulations on the same TK1–VIM pair, across 75 weight combinations, supports interpretation of the result as a structural property of the canonical network rather than an artefact of any single rule set.

**Absence of experimental validation.** We acknowledge explicitly that the proposed TK1–VIM therapeutic pair has not been validated in vitro or in vivo within the present study; the framework reported here is entirely computational. The orthogonal computational support from AlphaGenome is supportive but is not a substitute for wet-lab validation. A staged experimental programme would include (i) genetic loss-of-function studies in MDA-MB-231 and additional TNBC cell lines representative of the major molecular subtypes (BL1, BL2, M, MSL, LAR; e.g., HCC1937, MDA-MB-468, BT-549) using siRNA, shRNA, or CRISPR-Cas9 ablation of TK1 and VIM, individually and in combination; (ii) pharmacological perturbation with available inhibitors (3′-azido-3′-deoxythymidine and related nucleoside analogues for TK1; Withaferin A and FiVe1 for VIM) with formal IC50 determination and Bliss- or Loewe-based synergy quantification; (iii) phenotypic readouts of proliferation, apoptosis, migration and invasion appropriate to a mesenchymal-invasive phenotype; (iv) in vivo validation in patient-derived xenograft or syngeneic murine TNBC models, with attention to the tumor-microenvironment interactions discussed in Section 3.2.

**Generalizability across TNBC subtypes.** TNBC is molecularly heterogeneous, with at least four to six well-characterized subtypes (Lehmann classification: BL1, BL2, M, MSL, LAR) differing in transcriptomic profile and therapeutic vulnerability. Our analysis is based primarily on the mesenchymal-like MDA-MB-231 cell line; the identified TK1–VIM vulnerability is, therefore, most likely to generalize to other mesenchymal-like (M) or mesenchymal-stem-like (MSL) tumors, and may be less directly relevant to basal-like (BL1/BL2) or luminal-androgen-receptor (LAR) tumors that do not depend on the EMT axis. Extension of this computational framework to networks representative of other TNBC subtypes, and to patient-derived organoids or xenografts spanning the subtype spectrum, is a necessary next step.

**AlphaGenome limitations.** AlphaGenome is trained on bulk-tissue transcriptomic and epigenomic data and does not contain a TNBC-specific ontology. The mammary-tissue tracks used here represent normal mammary biology and aggregate cell-type heterogeneity within each compartment (in particular, our ‘mammary epithelium’ pool includes basal-myoepithelial and stem-cell populations that physiologically express some mesenchymal markers, partially elevating the apparent VIM baseline relative to a hypothetical pure luminal-epithelial sample). For these reasons, the AlphaGenome results in this work should be interpreted as supportive orthogonal computational evidence rather than as functional validation of in vivo behavior in TNBC.

### 3.5. Clinical Implications and Druggability

The identification of TK1–VIM as a high-confidence computationally nominated therapeutic pair has significant translational implications. A key advantage of this pair is its 100% perturbability—that is, both targets have existing pharmacological inhibitors, which we term “computationally druggable” to distinguish the availability of reported inhibitors from established clinical druggability in TNBC combination therapy, which would require formal pharmacokinetic, synergy, and toxicity evaluation. For TK1, compounds such as 3′-azido-3′-deoxythymidine (AZT) and other nucleoside analogs have established inhibitory effects; isoform-selective TK1 inhibitors are an active area of medicinal-chemistry development, given the need to minimize off-target effects on the closely related mitochondrial thymidine kinase TK2. For VIM, specific inhibitors like Withaferin A (WFA), a steroidal lactone from *Withania* somnifera that binds covalently to Cys328 in the vimentin rod domain and inhibits cancer-cell invasion and metastasis [33,34] and FiVe1 (FOXO3-induced Vimentin effector 1), a small molecule identified from a synthetic-lethal screen that selectively and irreversibly inhibits the growth of mesenchymally transformed cancer cells by binding the VIM rod domain and triggering mitotic catastrophe [35], have demonstrated efficacy in disrupting vimentin networks and impairing cancer cell motility in preclinical models; current limitations include moderate potency, suboptimal pharmacokinetic profiles, and limited clinical-grade chemistry.

To our knowledge, simultaneous pharmacological inhibition of TK1 and VIM has not been previously reported in TNBC. The computational rationale derived here, therefore, identifies a novel combinatorial strategy. While these inhibitors are primarily in preclinical or early clinical stages for solid tumors, their availability facilitates immediate experimental validation and provides a clear translational path. Future clinical translation will require careful evaluation of the combined toxicity profile, but the complementary mechanisms of action—targeting proliferation and metastasis simultaneously—offer a promising strategy for improving patient outcomes in TNBC.

## 4. Materials and Methods

### 4.1. Problem Formulation

The primary objective of this work is to identify a minimal set of therapeutic targets within a gene regulatory network that maximizes the transition of cancer cells from a malignant phenotype to an apoptotic one. We formalize this as follows:

Let G=(V,E) be a directed gene regulatory network where *V* is the set of genes (|V|=131) and *E* is the set of regulatory interactions. Each gene i∈V has a binary state $s_{i}\in \left\{0,1\right\}$ at time *t*, where 1 denotes active expression and 0 denotes inactive expression. The network dynamics are governed by Boolean update functions $f_{i}:{\left\{0,1\right\}}^{|R_{i}|}\to \left\{0,1\right\}$, where $R_{i}$ is the set of regulators of gene *i*.

Let $A_{0}$ denote the attractor representing the malignant phenotype (Attractor_0) and $A_{1}$ denote the attractor representing the apoptotic phenotype (Attractor_1). Our goal is to find an optimal target set $T^{*}\subseteq V$ with $|T^{*}|\leq k$ (where k=2) such that inhibiting the genes in $T^{*}$ causes the network to transition from the malignant state to a state maximally similar to the apoptotic phenotype.

Formally, the optimization problem is

(1) $$T^{*}=\underset{T\subseteq V,|T|\leq k}{\mathrm{argmax}}\;\mathrm{Similarity}\left(\mathrm{Simulate}\left(A_{0},T\right),A_{1}\right)$$

where $\mathrm{Simulate}(A_{0},T)$ returns the attractor state reached after Boolean simulation starting from the malignant state $A_{0}$ with genes in *T* held at state 0 (inhibited), and Similarity(·,·) measures the Hamming similarity between two states (defined in Equation (8)). The solution must satisfy additional constraints: high statistical robustness across multiple initial conditions, insensitivity to model parameter variations, and high druggability (availability of specific inhibitors for both targets).

### 4.2. Data and Network Construction

We utilized the exact 131-gene regulatory network from Sgariglia et al. (2024) [9], constructed from the MDA-MB-231 triple-negative breast cancer cell line (RRID:CVCL_0062). The network is a directed graph G=(V,E), where *V* is the set of 131 genes (|V|=131) and *E* is the set of 29 curated regulatory interactions (Supplementary Table S1). The 29 edges encode canonical TNBC signaling axes (STAT3 transcriptional program; PI3K/AKT survival signaling; HSP90 chaperone network; p53/MDM2 axis; intrinsic apoptosis cascade; NF-κB inflammation/survival; HIF1α hypoxic response; cell cycle regulation via CCND1; TGFβ/SMAD2 EMT axis; and HDAC1-mediated transcriptional repression) and were curated from the primary literature by Sgariglia et al. (2024) [9]. The Boolean update functions are modeled as nested canalizing functions, as defined by Kauffman et al. (2004) [32]. This network has been experimentally validated and is publicly available in the Supplementary Materials of the original publication.

### 4.3. Attractor Landscape and Phenotypic States

The published work by Sgariglia et al. (2024) [9] identified three stable attractors representing distinct cellular phenotypes through analysis of 30 independent samples from the MDA-MB-231 cell line:

- **Attractor_0 (Malignant Phenotype):** Characterized by high proliferation, survival gene activation, and low apoptosis markers. This represents the unperturbed cancer cell state.
- **Attractor_1 (Apoptotic Phenotype):** Characterized by cell cycle arrest, apoptosis gene activation, and survival gene inhibition. This represents the desired therapeutic outcome.
- **Attractor_2 (Alternative Phenotype):** A secondary stable state with intermediate characteristics.

The binary gene states for each attractor across all 30 samples are publicly available as Supplementary Table S2 in Sgariglia et al. (2024) [9] and form the basis of our analysis. For each sample s∈{1,2,…,30}, we have state vectors $A_{0}^{\left(s\right)},A_{1}^{\left(s\right)},A_{2}^{\left(s\right)}\in {\left\{0,1\right\}}^{131}$ representing the gene states in each attractor.

### 4.4. Gene Variability Analysis and Candidate Selection

To identify genes with high potential to influence attractor transitions, we calculated a variability score for each gene. For each gene i∈V, the variability score Var(i) quantifies the number of state changes across the three attractors and 30 samples:

(2) $$\mathrm{Var}\left(i\right)=\sum_{s=1}^{30}\left[⊮\left(A_{0,i}^{\left(s\right)}\neq A_{1,i}^{\left(s\right)}\right)+⊮\left(A_{1,i}^{\left(s\right)}\neq A_{2,i}^{\left(s\right)}\right)+⊮\left(A_{2,i}^{\left(s\right)}\neq A_{0,i}^{\left(s\right)}\right)\right]$$

where ⊮(·) is the indicator function. Genes with high variability are critical nodes for attractor transitions and, thus, strong therapeutic candidates. This analysis confirmed that VIM and TK1 exhibited the highest variability (20 differences each across samples and attractors), making them primary candidates for therapeutic targeting.

### 4.5. Target Identification via Semidefinite Programming

To identify nodes with the greatest potential to influence the transition from the malignant to the apoptotic phenotype, we formulated the problem using three complementary SDP optimizations. SDP is a subfield of convex optimization that provides tractable relaxations for NP-hard combinatorial problems, offering more robust solutions than purely heuristic methods [36,37].

**Formulation 1—Max-Cut SDP:** The objective is to partition the network nodes to maximize the weight of edges connecting survival and apoptosis gene groups, using the Goemans–Williamson SDP relaxation [36] (Equation (3)):

(3) $$\begin{array}{cccc} \; & \underset{X}{\mathrm{maximize}} & \; & \frac{1}{4}\sum_{i,j}W_{ij}\left(1-X_{ij}\right)\\ \; & \mathrm{subject}\;\mathrm{to} & \; & X_{ii}=1,\;\forall i\in V\\ \; & \; & & X⪰0 \end{array}$$

where *X* is the positive semidefinite variable matrix and *W* is the weighted adjacency matrix.

**Formulation 2—Influence Maximization SDP:** We seek to identify a set of “seed” nodes that maximizes the propagation of pro-apoptotic signals (Equation (4)):

(4) $$\begin{array}{cccc} \; & \underset{x}{\mathrm{maximize}} & \; & \sum_{j\in G_{A}}x_{j}+\sum_{i\in V}\sum_{j\in G_{A}}P_{ij}x_{i}-\lambda \sum_{i\in G_{S}}x_{i}\\ \; & \mathrm{subject}\;\mathrm{to} & \; & \sum_{i}x_{i}\leq k,\;0\leq x_{i}\leq 1,\;\forall i\in V \end{array}$$

where $x_{i}$ is the probability of selecting node *i*, $P_{ij}$ is the influence propagation probability, $G_{S}$ and $G_{A}$ are the sets of survival and apoptosis genes, λ is a penalty term, and *k* is the maximum number of targets.

**Formulation 3—Spectral Clustering SDP:** We use spectral clustering based on the network’s Laplacian matrix L=D−W (Equation (5)):

(5) $$\begin{array}{cccc} \; & \underset{Y}{\mathrm{minimize}} & \; & \mathrm{Tr}(LY)\\ \; & \mathrm{subject}\;\mathrm{to} & \; & Y_{ii}=1,\;\forall i\in V,\;Y⪰0 \end{array}$$

The scores from the three formulations are normalized (min–max to [0,1]) and combined into a single SDP score for each gene *i*, as shown in Equation (6):

(6) $$S_{\mathrm{SDP}}\left(i\right)=w_{1}S_{\mathrm{MaxCut}}\left(i\right)+w_{2}S_{\mathrm{Influence}}\left(i\right)+w_{3}S_{\mathrm{Spectral}}\left(i\right)$$

where $w_{1}+w_{2}+w_{3}=1$. We used baseline weights of 0.4, 0.4, and 0.2, and performed a comprehensive sensitivity analysis on these parameters across 75 different weight combinations spanning the 2-simplex.

#### SDP Optimization Pipeline—Pseudocode

The integration of the three SDP formulations into a single combined target score proceeds as follows.

1. Construct weighted adjacency matrix *W* from *E* (positive for activation, negative for inhibition).
2. Solve Max-Cut SDP relaxation (Equation (3)) via CVXPY with SCS solver → $S_{\mathrm{MaxCut}}\left(i\right)$ for each i∈V.
3. Solve Influence Maximization SDP (Equation (4)) under sparsity constraint $\sum_{i}x_{i}\leq k$→$S_{\mathrm{Influence}}\left(i\right)$ for each i∈V.
4. Solve Spectral Clustering SDP on Laplacian L=D−W (Equation (5)) → $S_{\mathrm{Spectral}}\left(i\right)$ for each i∈V.
5. Min–max normalize each score vector to [0,1].
6. Compute combined score $S_{\mathrm{SDP}}\left(i\right)$ as in Equation (6).
7. Select top-*k* genes by $S_{\mathrm{SDP}}$ and validate by Boolean simulation (Section 4.6).
8. Repeat steps 5–7 over a grid of $\left(w_{1},w_{2},w_{3}\right)$ on the 2-simplex (75 combinations) for sensitivity analysis.

**Figure 8:**
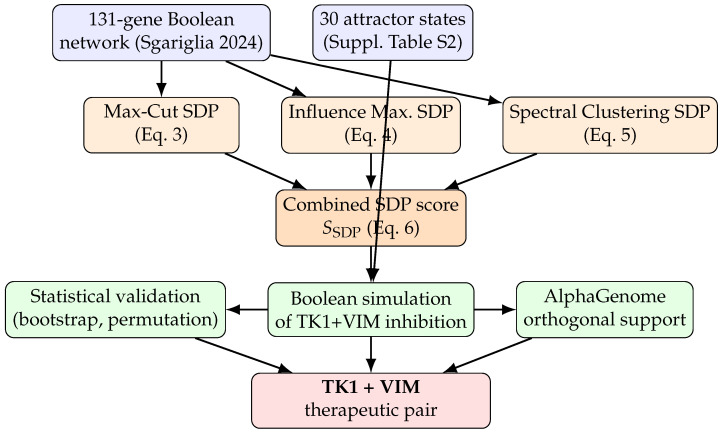
Computational pipeline. The 131-gene curated Boolean network feeds three complementary SDP formulations (orange), whose normalized scores are combined into a single ranking. The top-ranked pair is validated by Boolean dynamic simulation, statistical perturbation, and orthogonal AlphaGenome support (green), converging on TK1 + VIM as the proposed therapeutic pair [9].

The Python (Version 3.12.10) implementation is publicly available; SCS solver tolerances were left at default values, and the optimization pipeline runs in approximately five minutes on a standard workstation (16 GB RAM, no GPU required). The complete pipeline is summarized in Figure 8.

### 4.6. Functional Validation via Boolean Simulation

To validate the efficacy of a candidate target pair $T=\{t_{1},t_{2}\}$, we performed Boolean simulations. The network dynamics were modeled as a discrete-time system where the state of each gene $s_{i}\left(t+1\right)$ is updated based on the states of its regulators at time *t*, following the nested canalizing functions defined by Sgariglia et al. [9,32] (Equation (7)):

(7) $$s_{i}\left(t+1\right)=\left\{\begin{array}{cc} 0 & \mathrm{if}\;i\in T\\ f_{i}\left(s_{j_{1}}\left(t\right),s_{j_{2}}\left(t\right),\ldots \right) & \mathrm{otherwise} \end{array}\right.$$

where $f_{i}$ is the Boolean update function for gene *i* and $j_{k}$ are its regulators.

We simulated the network starting from the 30 malignant attractor states, with the target pair inhibited. The final state of each simulation was compared to the corresponding apoptotic attractor state to calculate three key metrics:

- **Attractor Similarity:** The percentage of genes in the final simulated state that match the apoptotic attractor state (Equation (8)).
  (8) $$\mathrm{Similarity}=\frac{1}{\left|V\right|}\sum_{i\in V}\left(1-\right|s_{i}^{\mathrm{final}}-s_{i}^{\mathrm{apoptotic}}\left|\right)$$
- **Survival Gene Inhibition:** The percentage of key survival genes (e.g., BCL2, MCL1) that are successfully inhibited (state 0) in the final state.
- **Apoptosis Gene Activation:** The percentage of key apoptosis-promoting genes (e.g., BAX, CASP3) that are successfully activated (state 1) in the final state.

### 4.7. Statistical Validation and Robustness Analysis

To ensure the robustness of our findings, we performed bootstrap resampling (*n* = 10,000) [38] on the 30 initial states to calculate 95% confidence intervals (CIs) for all validation metrics. To compare the performance of our identified pair (TK1–VIM) against five alternative pairs, we used paired *t*-tests and non-parametric permutation tests (*n* = 10,000) [39], with Bonferroni correction for multiple comparisons [40]. Alternative false-discovery rate methods (Benjamini–Hochberg [41]; Storey q-value [42]) were evaluated and yielded qualitatively identical conclusions; Bonferroni correction is reported as it provides the most conservative family-wise error rate control appropriate for a small number of prespecified comparisons. Specifically, the five comparisons (TK1–VIM versus STAT3–BCL2L1, AKT2–MDM2, HSP90AB1–NFKB1, TP53–BAX, and CCND1–E2F1) were corrected at α = 0.05/5 = 0.01; all observed *p*-values remained below 0.001 after correction. Cohen’s *d* was calculated to quantify the effect size [43]; observed values of d=5.28–5.45 correspond to extremely large effects (conventional “large” threshold d>0.8). The 30 paired observations correspond to distinct biological replicates of the MDA-MB-231 cell line in the originally published dataset [9], supporting the assumption of sample independence.

### 4.8. Orthogonal Computational Support via AlphaGenome

To provide an orthogonal layer of computational support, we used AlphaGenome [30], a large-scale deep learning model trained on vast genomic and transcriptomic datasets, to predict the genome-wide effects of inhibiting TK1 and VIM in normal mammary tissue. We queried the model for predicted RNA-seq expression and for the effect of single-nucleotide variants 300 bp upstream of each gene’s transcription start site, across a 524,288 bp (512 kb) interval centered on TK1 (chr17:7,577,845; genome build GRCh38) and VIM (chr10:17,270,258; GRCh38). Ontology terms were validated against the AlphaGenome catalogue of 704 supported terms via a dedicated preflight script (Supplementary Code S6) prior to any analytical query.

The mammary-tissue ontology terms were partitioned a priori into two compartments (mammary epithelium, 5 terms/13 tracks; mammary stroma, 3 terms/2 tracks; see Section 2.6 for full term lists). Four reference tissues (liver UBERON:0002107; skeletal muscle UBERON:0001134; brain UBERON:0000955; heart UBERON:0000948) were queried as off-target organ comparators. Compartment- and tissue-specific means were computed by averaging across all returned tracks. The complete query log, including timestamp, ontology terms, and per-track values, is provided as Supplementary Data alphagenome_v3_results.json; the ontology audit log is provided as Supplementary Table S5.

The convergence of results from these two fundamentally different computational paradigms—a curated, knowledge-driven Boolean model and a data-driven, large-scale deep learning model—provides orthogonal computational support for the biological plausibility of the predicted therapeutic effects, without substituting for experimental validation.

## 5. Conclusions

We have demonstrated that a hybrid computational approach combining Boolean network modeling, semidefinite programming optimization, statistical validation, and deep learning support can identify robust therapeutic targets for TNBC. The TK1–VIM pair emerges as a high-confidence, druggable therapeutic strategy with strong biological plausibility. The compartment-stratified AlphaGenome analysis revealed a biologically coherent pattern in which VIM is enriched 2.2-fold in mammary stroma over mammary epithelium, consistent with its role as a mesenchymal marker, and in which simulated inhibition of either target induces near-zero transcriptomic perturbation of normal mammary epithelium (mean $|\log_{2}\mathrm{FC}|<0.001$). This provides a clear biological rationale for synergistic targeting: TK1 inhibition attacks the proliferative capacity of the bulk tumor, while VIM inhibition prevents the emergence of metastatic, invasive cell populations. This work establishes a rigorous methodological benchmark for computational drug target identification and provides a computationally grounded, druggable candidate pair as a starting point for experimental computational validation in TNBC treatment research.

The next concrete steps emerging from this work are (i) prospective experimental validation of dual TK1–VIM inhibition in a panel of TNBC cell lines representative of the molecular subtype spectrum, with formal synergy quantification (Bliss/Loewe); (ii) preclinical evaluation in patient-derived xenograft models with mesenchymal-like histology; (iii) extension of the hybrid SDP–Boolean framework to subtype-specific regulatory networks; and (iv) refinement of the computational pipeline to incorporate dose–response (IC50) representation and stochastic Boolean variants, addressing the binary-state limitation of the current model. Through these next steps, we aim to bridge the in silico-to-in vivo gap that remains the principal limitation of computational target identification.

Beyond the specific TK1–VIM finding, the model developed here is intended as a reusable, general-purpose framework. Because the pipeline is agnostic to the particular network supplied, it can be redeployed to other malignancies and regulatory contexts simply by substituting the input Boolean network and its attractor landscape: the SDP-based ranking, multi-formulation consensus, statistical robustness analysis, and orthogonal AlphaGenome support all transfer unchanged. Prospective applications of the developed model, therefore, include (a) systematic reanalysis of existing curated cancer Boolean networks to nominate minimal druggable target sets; (b) coupling the framework to automated network-inference methods so that patient-specific or subtype-specific networks can be generated and optimized in a single workflow; and (c) integration of the SDP ranking with druggability and toxicity priors as additional constraints, moving the model toward an end-to-end computational platform for combinatorial target discovery. We anticipate that this reusable design will allow the present methodology to serve as a benchmark and a practical tool for the broader computational drug-discovery community.   

## Figures and Tables

**Table 1:** Comparative analysis of methodological generations for TNBC target identification.

Feature	Gen 1 (Tilli et al. [8])	Gen 2 (Sgariglia et al. [9])	This Work (Gen 3)
Methodology	Heuristic (PPI)	Heuristic (Boolean)	Hybrid (SDP + Boolean)
Number of Targets	5	3	**2**
Druggability	40% (2 of 5)	67% (2 of 3)	**100% (2 of 2)**
Statistical Validation	None	None	**Yes (Bootstrap, Permutation Tests)**
Sensitivity Analysis	None	None	**Yes**
Orthogonal Computational Support	None	None	**Yes (AlphaGenome)**

**Table 2:** Validation metrics for TK1–VIM inhibition with 95% confidence intervals.

Metric	Mean Score	95% CI
Attractor Similarity	0.9903	[0.9879, 0.9926]
Survival Gene Inhibition	0.6601	[0.6424, 0.6778]
Apoptosis Gene Activation	0.5560	[0.5310, 0.5810]

**Table 3:** Statistical comparison of TK1–VIM vs. alternative pairs.

Comparison	Mean Diff.	Cohen’s *d*	*p*-Value (*t*-Test)	*p*-Value (Permutation)
TK1–VIM vs. STAT3–BCL2L1	0.254	5.28	<0.001	<0.001
TK1–VIM vs. AKT2–MDM2	0.251	5.38	<0.001	<0.001
TK1–VIM vs. HSP90AB1–NFKB1	0.274	5.45	<0.001	<0.001
TK1–VIM vs. TP53–BAX	0.291	5.41	<0.001	<0.001
TK1–VIM vs. CCND1–E2F1	0.325	5.33	<0.001	<0.001

**Table 4:** AlphaGenome predictions for TK1 and VIM in normal mammary tissue, stratified by compartment. Values are normalized RNA-seq units averaged across all available tracks in each ontology pool.

Compartment	Ontology Term Pool	n Tracks	TK1 Mean	VIM Mean	TK1/VIM
Mammary epithelium	CL:0002327, UBERON:0008367, CL:0002324, CL:0002326, CL:0002451	13	0.159	0.143	1.12×
Mammary stroma	CL:0002555, CL:2000071, NTR:0003079	2	0.146	0.317	0.46×
*Stroma/Epithelium ratio*			0.92×	**2.22**×	—

## Data Availability

The Boolean network model and attractor state data are available in the Supplementary Materials of Sgariglia et al. (2024) [9]. AlphaGenome predictions are provided as Supplementary Data (alphagenome_v3_results.json). The preflight ontology-verification script and the corrected analysis script are provided as Supplementary Code. All code used for SDP optimization, statistical analysis, and AlphaGenome integration has been deposited in a public Zenodo repository with a persistent DOI [6]: https://zenodo.org/records/20476101 (doi:10.5281/zenodo.20476101; accessed on 11 June 2026). The repository contains Boolean network scripts, SDP pipeline (CVXPY/SCS), statistical analysis routines, AlphaGenome query scripts, and the full AlphaGenome v3 raw output.

## Supplementary Materials

The following supporting information can be downloaded at: https://www.mdpi.com/article/10.3390/ijms27125385/s1.

## Abbreviations

The following abbreviations are used in this manuscript:

TNBC	Triple-negative breast cancer
SDP	Semidefinite programming
TK1	Thymidine Kinase 1
VIM	Vimentin
EMT	Epithelial–mesenchymal transition
CI	Confidence interval
DALY	Disability-adjusted life year
IHC	Immunohistochemistry
IC50	Half-maximal inhibitory concentration
TME	Tumor microenvironment
RRID	Research Resource Identifier
OLS	Ontology Lookup Service (EBI)
UBERON	Uber Anatomy Ontology
CL	Cell Ontology
